# The Impact of Ten Days of Periodic Fasting on the Modulation of the Longevity Gene in Overweight and Obese Individuals: A Quasi-Experimental Study

**DOI:** 10.3390/nu16183112

**Published:** 2024-09-15

**Authors:** Nurma Yuliyanasari, Eva Nabiha Zamri, Purwo Sri Rejeki, Muhammad Miftahussurur

**Affiliations:** 1 Doctoral Programs of Medical Science, Faculty of Medicine, Universitas Airlangga, Surabaya 60113, Indonesia; nurma.yuliyanasari-2021@fk.unair.ac.id; 2 Department of Physiology, Faculty of Medicine, Universitas Muhammadiyah Surabaya, Surabaya 60132, Indonesia; 3 Department of Community Health, Advanced Medical and Dental Institute, Universiti Sains Malaysia, Kepala Batas, Pulau Pinang, Bertam 13200, Malaysia; evazamri@usm.my; 4 Centre for Epidemiology and Evidence-Based Practice, Department of Social and Preventive Medicine, University of Malaya, Kuala Lumpur 50603, Malaysia; 5 Physiology Division, Department of Medical Physiology and Biochemistry, Faculty of Medicine, Universitas Airlangga, Surabaya 60132, Indonesia; 6 Division of Gastroenterology-Hepatology, Department of Internal Medicine, Faculty of Medicine-Dr. Soetomo Teaching Hospital, Universitas Airlangga, Surabaya 60286, Indonesia; 7 *Helicobacter pylori* and Microbiota Study Group, Institute Tropical Disease, Surabaya 60115, Indonesia

**Keywords:** FOXO, h-TERT, obesity, periodic fasting, telomerase

## Abstract

Background: Fasting potentially alters the aging process induced by obesity by regulating telomere integrity, which is related to longevity genes. However, the impact of periodic fasting (PF) on the expression of longevity genes, particularly Forkhead Box O Transcription Factors (FOXO3a) and the Human Telomerase Reverse Transcriptase (hTERT), is not fully understood. This study aimed to analyze the effects of PF, specifically on FOXO3a, hTERT expression, and other associated factors. Methods: A quasi-experimental 10-day study was conducted in Surabaya, East Java, Indonesia. This study consisted of an intervention group (PFG), which carried out PF for ten days using a daily 12 h time-restricted eating protocol, and a control group (CG), which had daily meals as usual. FOXO3a and hTERT expression were analyzed by quantitative real-time qPCR. A paired *t*-test/Wilcoxon test, independent *t*-test/Mann–Whitney U-test, and Spearman’s correlation test were used for statistical analysis. Result: Thirty-six young men participated in this study. During the post-test period, FOXO3a expression in the PFG increased 28.56 (±114.05) times compared to the pre-test, but the difference was not significant. hTERT expression was significantly higher in both the CG and PFG. The hTERT expression in the PFG was 10.26 (±8.46) times higher than in the CG, which was only 4.73 (±4.81) times higher. There was also a positive relationship between FOXO and hTERT in the CG. Conclusions: PF significantly increased hTERT expression in the PFG; however, no significant increase was found in FOXO3a expression. PF regimens using the 12 h time-restricted eating approach may become a potential strategy for preventing obesity-induced premature aging by regulating longevity gene expression.

## 1. Introduction

Aging is a complex process requiring global attention. A continuous decrease due to a series of cellular and molecular damages can eventually cause impairment in physical function and a higher risk of some aging-related diseases [1,2]. Aging-related disease is an ongoing health condition that is common, costly, and a leading cause of mortality and disability among the elderly [3]. Unfortunately, this condition tends to be identified at a younger age. In 2019, the most common chronic disease among young men in the USA was obesity (25.5%) [4]. Data from the WHO showed that there were 2.5 billion overweight adults and more than 890 million obese adults in 2022 [5]. Recently, obesity has not only lead to various health disorders, but has also accelerated aging by affecting genome maintenance and stability [6,7,8,9].

Telomeres are known for maintaining genomic integrity by protecting the chromosomes from DNA damage and end-to-end fusion. Unfortunately, as we age, telomere length naturally decreases to a minimal critical length [10]. Similar to aging, obesity is also associated with telomere function, integrity, and length [11]. Obesity can cause oxidative stress and inflammation, which may advance the shortening of telomeres [12]. As a result, there must be an approach to maintain telomere integrity in individuals with obesity.

A reduction in telomerase, an enzyme that protects telomeres, is one of the causes of greater telomere shortening along with age. Its activity is regulated by various factors, including the Human Telomerase Reverse Transcriptase (hTERT) gene. hTERT is presumed to contribute to aging and has been used as a molecular marker for cellular senescence [13]. One of the causes of hTERT expression is an increase in Forkhead Box O Transcription Factors (FOXOs), a family of transcription factors involved in aging and longevity [14,15]. FOXO3a plays a crucial role in some functions, such as regulating PGC-1α, apoptosis, inflammation, cell survival, proteostasis, autophagy, mitophagy, and stem cell function, which are all linked to aging. The role of FOXO on telomere integrity remains to be further studied; however, a study revealed that FOXO3a increased hTERT expression by activating c-MYC and increasing the replication lifetime of human fibroblasts [16].

In addition to preventing early aging induced by obesity, a therapeutic approach to affect longevity genes is more helpful [17]. Dietary interventions can modulate aging due to cellular and molecular mechanisms [18]. Intermittent fasting (IF) is one treatment that can slow the aging process and treat obesity [19]. One type of IF that might be interesting is periodic fasting (PF), a type of IF in which the individual must fast for a specific period (1–21 days), or longer [19,20,21]. This fasting type is often observed in many religions and is used as a medical intervention for metabolic diseases, such as obesity [22,23]. Previous studies have explained that 4–21 days of PF is safe and can improve health and well-being [21].

A previous study indicated that dietary control can decrease the aging and obesity phenotype by regulating FOXO [24]. Dietary approaches can inhibit the insulin/IGF-1 signaling (IIS) pathway, decrease Akt activity, and activate FOXO. FOXO activation can affect metabolic homeostasis, redox balance, and stress responses, all of which contribute to obesity phenotype and telomere integrity, which play an essential role in aging [24,25]. The relationship between PF, FOXO, and telomere function, especially the role of hTERT, is exciting to study further. This study aims to analyze the effects of PF on longevity genes, especially FOXO and hTERT expression, in young men with overweight and obesity. If these studies can be confirmed, then PF can be advised as an approach/adjuvant strategy to overcome obesity and prevent the early aging that will follow.

## 2. Materials and Methods

### 2.1. Study Design

**Figure 1:**
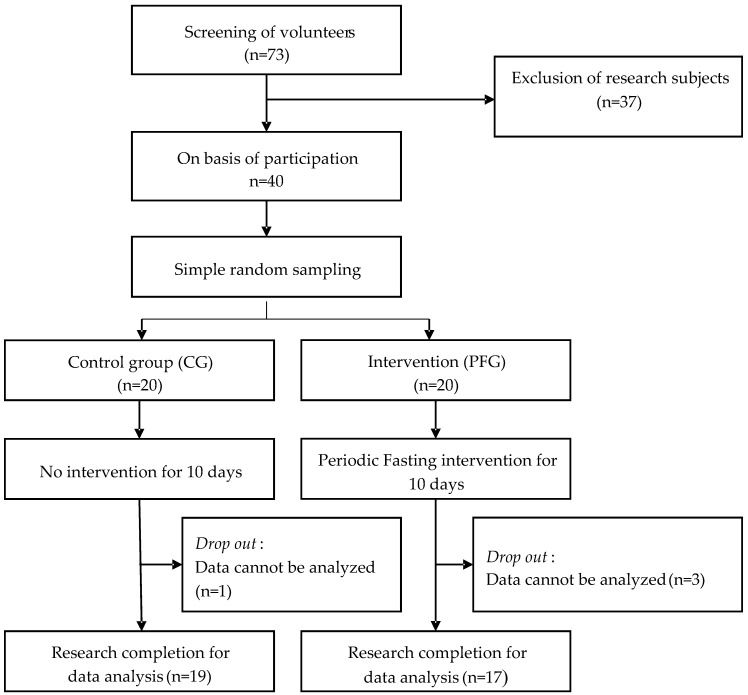
Research workflow.

This study is a quasi-experimental design with randomization in Surabaya, East Java, Indonesia. Two groups participated in this study—the control group (CG), which did not receive any kind of treatment, and the treatment group (PFG), which carried out PF for 10 days. In Figure 1, the research workflow is displayed.

### 2.2. Participants

Men with an age ≥ 20, a good overall health, and with a BMI that is in the overweight (23–24.9 kg/m^2^) and obese ((BMI ≥ 25 kg/m^2^) category according to the guidelines for Asians and light-to-moderate physical activity (<600 MET or 600–3000 MET) as measured by the Global Physical Activity Questionnaire (GPAQ) participated in this study [26,27,28,29,30]. This study excluded those presently participating in weight-loss programs or following other eating patterns, such as vegetarianism or veganism, to establish the intervention’s validity. In addition, diseases or conditions that alter physiology, hormones, and metabolism were excluded, such as a history of diabetes mellitus, thyroid and parathyroid problems, heart illnesses, cancer, drunkenness, smoking, daily use of acetylsalicylate medicines, or usage of hormonal prescriptions [31]. The 40 participants who met the inclusion conditions were split into two groups using simple random sampling. Randomization was performed by assigning numbers to each participant and randomly assigning them into one of two groups.

### 2.3. Data Collection

Study parameters were measured by professional laboratory assistants in a physiology laboratory both before and after treatment to collect research data. After completing the screening procedure, participants were invited to a coordination group to help decide upon the test schedule and venue. Data were collected before the patient had breakfast, from 06:00 to 09:00 a.m., in the physiology laboratory of Universitas Muhammadiyah Surabaya and Airlangga. The patient was asked not to eat for at least eight hours before the test.

### 2.4. Periodic Fasting Protocol

Participants in the CG were requested to have daily meals as usual. Contrastingly, participants in the PFG were asked to use a time-restricted eating regimen of a 12 h fast each day for ten days. The participants had two meals—one in the morning before 06:00 a.m. and another in the evening after 06:00 p.m. All participants in the PFG received food with measured daily calories. Meals supplied by the authors were only allowed to be consumed by the PFG within ten days [19,21,23,32]. Monitoring cards were used in this study to monitor food intake and regulate conditions within the group. The CG and PFG received encouragement to comply with their present diet, exercise, and physical activity routines.

### 2.5. Physiological Parameter Measurements

#### 2.5.1. Vital Sign Parameter Measurements

Vital sign parameters consist of three measurements such as systolic blood pressure (SBP), diastolic blood pressure (DBP), respiration rate (RR), and heart rate (HR). A one-minute rest period was allowed between each measurement of SBP and DBP, which were taken on the non-dominant arm using an Omron Tensimeter Digital HEM 7156 T (Omron Healthcare Manufacturing Vietnam Co., Ltd., Binh Duong, Vietnam).

#### 2.5.2. Anthropometric and Body Composition Parameter Measurements

This study measured anthropometric parameters like body weight (BW), body height (BH), abdominal circumference (AC), waist circumference (WC), and body mass index (BMI). Standing measurements were made for anthropometric measures such as BW and BH, which were reported using scales (Zhongshan Camry Electronic Co., Ltd., Guangdong, China) to the nearest 0.5 kg. BMI was calculated by dividing body weight by height squared (kg/m^2^) using standards from Asia-Pacific [30]. Body composition provides estimates of body fat (BF), muscle mass (MM), total body water (TBW), visceral fat level (VFL), and bone mass (BM), which were quantified using bioelectrical impedance analysis (BIA) Tanita BC-545N (Tanita Corporation, Tokyo, Japan) and were measured 2 times before and after PF treatment.

#### 2.5.3. Glucose and Lipids Profile Measurements

Fasting blood glucose (FBG) and total cholesterol (TC) levels were measured using a glucometer (Zhongshan CAMRY Electronic Co., Ltd., Jhunan Twonship, Taiwan).

### 2.6. Blood Sample Collection and Storage

All participants had their antecubital veins used to draw blood samples. Up to 7 ccs of blood were permitted to run through the vacutainer and were then put into an additive-free tube containing ethylene diamine tetra acetic acid. After that, tubes containing blood samples were shipped on dry ice to the laboratory [33,34]

### 2.7. Peripheral Blood Mononuclear Cell (PMBC) Extraction

PBMC extraction was performed by centrifuging blood samples at 2000 rpm for 10 min. The plasma was then transferred into a tube (temperature kept at −80 °C), and the remaining material in the conical tube was mixed with 3 mL of Dulbecco’s phosphate-buffered saline (PBS) 1× (Cat. No 17-1440-02, Sigma, Ltd., Kawasaki-shi, Japan) and 5 mL of Ficoll-Paque PLUS (Cat no.17-14440-02, Cytiva, Marlborough, MA, USA). The solution was combined and centrifuged at 1700 rpm for 30 min. The upper liquid was discarded, while the intermediate liquid was transferred to a new conical tube with middle liquid supplemented with PBS 1× to 15mL. The samples were centrifuged at 1500 rpm for 5 min. The upper liquid was removed and 1× 15 mL of PBS was added. The sample was then spun at 1300 rpm for 5 min. After discarding the liquid in the sample, 600 µL of 1× PBS were added and mixed with a pipette. The material was then transferred to a 1.5 mL tube and stored at −30 °C [33,34].

### 2.8. RNA and DNA Extraction

RNA extraction was performed on the samples using the QIAamp RNA Blood Mini Kit (Cat. 52304, Qiagen, Valencia, CA, USA) according to the manufacturer’s protocol. The obtained RNA was then converted into first-strand cDNA with the GoScript™ Reverse Transcription System (Cat. No. A5000, Promega, Madison, WI, USA). cDNA was counted using GoTaq^®^ qPCR Master Mix (Cat. No. A6001, Promega, USA). FOXO expression was quantified using qPCR and primers FOXO3a (Macrogen, Seoul, Republic of Korea). The primer sequence used was F(5′-CTTCAAGGATAAGGGCGACAG-3′)/R(5′-TCGTCCTGGACTTCATCCAAC-3′) [33]. Glyceraldehyde 3-phosphate dehydrogenase (GAPDH), a housekeeper gene, sequences were F(5′-CAATGACCCCTTCATTGACC-3′/R: 5′-GAAGATGGTGATGGGATTTC-3′). These primers were blasted using primer-BLAST on the NCBI website. Thermal cycling was performed with GoTaq^®^ Hot Start Polymerase, with an initial denaturation (hold) at 95 °C for 120 s, 40 cycles of amplification at 95 °C for 10 s, 55 °C for 17 s, and 72 °C for 17 s, followed by melting at 60 °C for 60 s and 90 °C for 1 s.

According to the manufacturer’s instructions, DNA was extracted from whole blood using the QIAamp Blood Mini Kit (cat. nos. 51104 and 51106) (Qiagen, Valencia, CA, USA). DNA was counted using GoTaq^®^ qPCR Master Mix (Cat. No. A6001, Promega, USA). hTERT was quantified using qPCR; the hTERT primer sequence (Macrogen, Republic of Korea) was F(5′-CCCATTTCATCAGCAAGTTTGG-3′), R(CTTGGCTTTCAGGATGGAGTAG-3′ [35] and the GAPDH sequence was F(5′-CAATGACCCCTTCATTGACC-3′/R: 5′-GAAGATGGTGATGGGATTTC-3′). These primers were blasted using primer-BLAST on the NCBI website. Thermal cycling was performed with GoTaq^®^ Hot Start Polymerase, with an initial denaturation (hold) at 95 °C for 120 s, 40 cycles of amplification at 95 °C for 10 s, 55 °C for 17 s, and 72 °C for 17 s, followed by melting at 60 °C for 60 s and 90 °C for 1 s.

### 2.9. FOXO and hTERT Expression Analysis

The expression of FOXO3 was determined using the Livak method (2^−ΔΔCq^) and was written as a ratio (a relative expression) [34,36]. The post-test expression of FOXO mRNA was then compared with that of the pre-test. The relative mRNA expression level of FOXO3a was normalized to the geometric mean of GAPDH. The expression of hTERT was also determined using the Livak method and was written as a ratio (a relative expression) [35,36]. To better describe the expression data, we will also display data based on the dCT value of each gene.

Note:

ΔΔCT = ΔCT(test) − ΔCT(calibrator)
ΔCT(test) = CT(target, test) − CT(reference, test)
ΔCT(calibrator) = CT(target, calibrator) − CT(reference, calibrator)
Targets = FOXO3, hTERT
TEST = CG and PFG
Calibrator = control group
Reference = GADPH

### 2.10. Statistical Analysis

The data were analyzed using the SPSS statistical program for social science (SPSS, Inc. Chicago, IL, USA). Continuous data were expressed as mean ± SD, whereas categorical variables were shown as numbers and percentages. The Kolmogorov–Smirnov test was used to assess the normality of the data; the Wilcoxon and paired *t*-tests were used to analyze the differences between the pre-test and post-test; the independent *t*-test/Mann–Whitney test was used to determine group differences. The Pearson or Spearman correlation test was used to analyze the correlation among the variables.

## 3. Results

### 3.1. Physiological Characteristics of Participants

#### 3.1.1. Age and Physical Activity of Participants

This study included 40 out of 73 individuals who met the requirements; in total, 36 participants (90%) finished the study. Four participants were excluded because their data were too biased due to their characteristics. Of the participants, 19 and 17 were assigned to the CG and PFG, respectively. Figure 1 illustrates the research workflow. In this study, participants in the CG and PFG had a mean age of 20.89 (±1.05) and 20.59 (±0.94), respectively. The CG and PFG engaged in moderate physical activity, with mean GPAQ scores of 1727.42 (±1013.35) MET and 1918.50 (±1099.17) MET, respectively. However, the difference in mean age and physical activity was not significant between both groups. There was no significant difference in all baseline characteristics at the pre-test time between both groups (all *p* > 0.05).

#### 3.1.2. Effects of PF on Vital Signs, and Anthropometric and Body Composition of Participants

Table 1 shows the results of participants’ vital signs, and anthropometric and body composition tests. After 10 days of monitoring, the CG showed no significant differences in pre-test and post-test physiological parameters, whereas the PFG showed significant differences in several parameters. In the PFG, the post-test value for SBP was observed to be significantly lower than the pre-test value (*p* < 0.05). After 10 days of PF, the PFG’s anthropometric and body composition parameters showed improvement, with significant decreases in BW, BMI, AC, WC, and FVL (*p* < 0.05). However, participants’ MM showed a substantial decrease (*p* < 0.05). Table 1 also compares differences (Δ) between the post-test and pre-test parameter results in the CG and PFG. The differences (Δ) in BW, BMI, AC, WC, and MM between the CG and PFG were statistically significant

#### 3.1.3. Effects of PF on Glucose and Lipid Profiles of Participants

**Figure 2:**
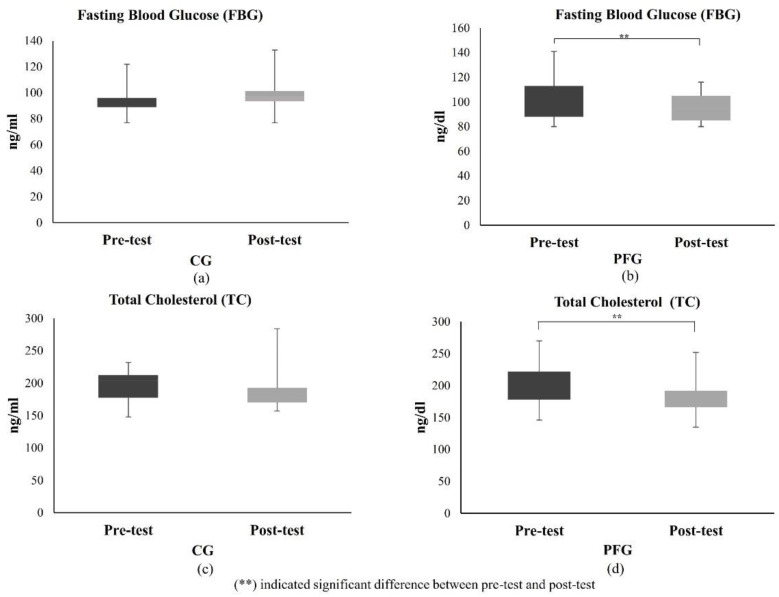
Effects of PF on glucose and lipid profiles after 10 days. (**a**) FBG level in the CG; (**b**) FBG level in the PFG; (**c**) TC level in the CG; (**d**) TC level in the PFG. The difference between pre-test and post-test data was analyzed using the paired *t*-Test or Wilcoxon test. (**) indicates a significant difference.

Figure 2 displays the results of the FBG and TC tests. Compared to the CG, the PFG’s post-test FBG levels were considerably lower than the pre-test (Figure 2a,b). The difference in FBG levels between the two groups was significant (*p* < 0.05). The TC test results decreased in both the CG and PFG (Figure 2c,d). However, the decline in the PFG was significant (*p* < 0.05), whereas that of the CG was not significant (*p* > 0.05).

### 3.2. Effects of PF on the Longevity Gene Expression in the CG and PFG

#### 3.2.1. Effect of PF on FOXO3a Expression

**Figure 3:**
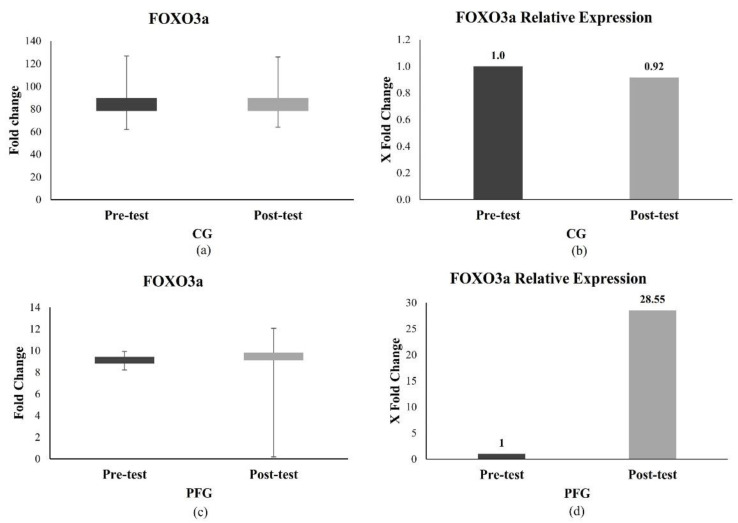
The difference in FOXO3a expression of the participants. (**a**) dCT of FOXO3a in the CG; (**b**) FOXO3a relative expression from ΔΔCt in the CG; (**c**) dCT of FOXO3a in the PFG; (**d**) FOXO3a relative expression from ΔΔCt in the PFG. The difference between pre-test and post-test data was analyzed using the paired *t*-test or Wilcoxon test.

Figure 3 shows the results of longevity-gene-related expression in PBMCs. Figure 3a,c show the dCT FOXO3a values in the CG and PFG. The mean dCT in the CG at pre-test and post-test was 8.91 (±0.80) and 9.35 (±1.28) (Figure 3a). Meanwhile, the mean dCT in the PFG at pre-test and post-test was 9.12 (±0.48) and 9.03 (±2.47), respectively (Figure 3c). Figure 3b,d show the relative expression (from ΔΔCt) of FOXO3 compared to controls (pre-intervention). It was found that the relative expression of FOXO in the CG was 0.92 times lower at post-test compared to pre-test (Figure 3b). The relative expression in the PFG was 28.56 times greater than in the pre-test. Despite the differences, there were no statistically significant differences between the CG and PFG (all *p* > 0.05) (Figure 3d).

#### 3.2.2. Effect of PF on hTERT Expression

**Figure 4:**
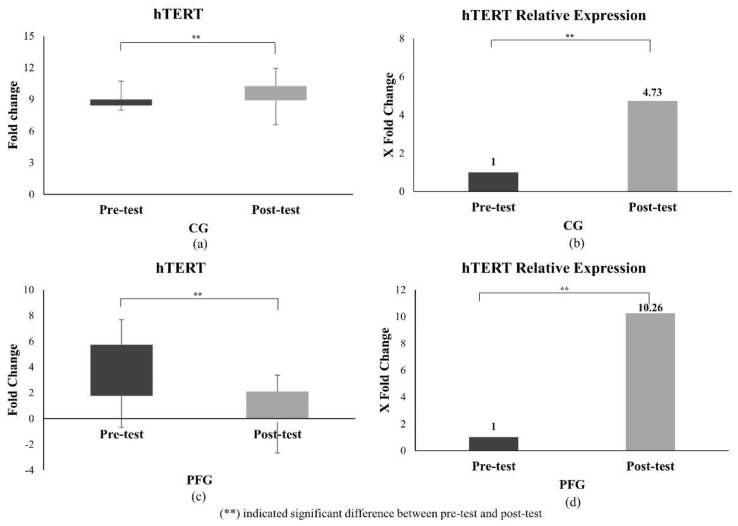
The difference in hTERT expression of the participants. (**a**) dCT of hTERT in the CG; (**b**) hTERT relative expression from ΔΔCt in the CG; (**c**) dCT of hTERT in the PFG; (**d**) hTERT relative expression from ΔΔCt in the PFG. The difference between pre-test and post-test data was analyzed using the paired *t*-test or Wilcoxon test. (**) indicates a significant difference.

hTERT is another longevity gene with significantly elevated relative expression in this study (Figure 4). Figure 4a,c show the dCT hTERT values in the CG and PFG. The mean dCT in the CG at pre-test and post-test was 3.06 (±1.65) and 2.14 (±2.76). Meanwhile, the mean dCT in the PFG at pre-test and post-test was 3.57 (±2.32) and 0.73 (±1.68), respectively. Figure 4b,d show the relative expression of hTERT compared to controls (pre-intervention) in the CG and PFG. The CG’s hTERT expression was 4.73 (±4.81) times higher at the post-test value than the pre-test value. At post-test, the relative expression of hTERT in the PFG was 10.26 (±8.46) times higher than the pre-test value. Both groups showed a significant increase in hTERT expression values (all *p* < 0.05), but the differences in the PFG were substantially higher compared to the CG (*p* < 0.05). Correlation analysis showed a significant moderate correlation between ΔFOXO and Δ hTERT in the CG (*p* = 0.00, r = 0.61), but no significant correlation was found in the PFG (*p* = 0.54, r = 0.16).

## 4. Discussion

Ten days of PF in our study can enhance some physiological characteristics of participants such as SBP, BW, BMI, AC, WC, MM, FVL, SBP, FBG, and TC. These conclusions were supported by the significant differences in changes between the CG and PFG, except for FVL. These findings were consistent with several studies showing that different IF approaches may improve weight loss and anthropometric parameters, while also enhancing cardiometabolic health in people with a higher BMI [37,38]. Theoretically, beyond the 12 h fasting state, blood glucose continues to slowly decline, which is in concordance with the reducing FBG in the PFG [38]. The reduction in FBG will continue by increasing fatty acid oxidation, causing a reduction in lipid profile in blood, such as low-density lipoprotein (LDL) or triglyceride [39]. This may explain the considerable decrease in TC in the PFG observed in our study.

The mean FOXO3a in the PFG was higher than that of the CG in our study, revealing that dietary modifications, such as IF, could enhance FOXO expression; however, not significantly. This was consistent with prior studies in rabbit liver, which found that IF significantly increased FOXO3 expression [35]. FOXO3a, a human longevity gene, may slow aging by increasing antioxidant expression and lowering ROS generation as a response to oxidative stress and cell injury associated with aging [15]. This gene would also be activated by participant telomere attrition, which, unfortunately, was not analyzed in this study.

An inadequate rise in FOXO in our study might occur as a result of various factors, for example, post-translational modifications (PTMs) such as phosphorylation, ubiquitination, acetylation, methylation, and other various external stimuli such as growth factor signaling, oxidative stress, genotoxic stress, and food deprivation [40,41]. Furthermore, a study showed that reduced telomere attrition might be a significant mechanism for the longevity-promoting impact of the investigated FOXO3 genotype. This effect was especially significant in older subjects (>50 years of age) [42,43]. Most of the participants in this study were still 20 years old, which might explain the lack of a visible difference in FOXO expression.

This study found that 10 days of PF could significantly increase hTERT expression, a telomerase regulatory gene, in the PFG. However, a similar study in humans remained limited. As a result, the decrease in FBG caused by the PF (Figure 2) in our study might be similar to that observed in diabetic rats fed a soy extract diet. Decreased FBG could increase the expression of the TERT protein in the islets of Langerhans in rats [44]. This result was further supported by a study conducted by Ali et al. (2016), which found that in nutrient-deprived situations, increased TERT function inhibits the kinase activity of mTOR complex 1 (mTORC1) in various cell lines, leading to the activation of autophagy [45]. This concept is similar to the PF principle, which can also improve autophagy by inhibiting mTORC1 [46,47].

However, the findings in this study differed from those of another study on cancer. In breast cancer patients, glucose restriction reduces the telomerase activity and mRNA expression of the catalytic component hTERT and enhances apoptosis via BIBR 1532-mediated telomerase suppression [48]. Due to the possibility of the varying regulation of hTERT in normal and malignant cells, the generated effect of diet modulation might also be different. hTERT is not expressed in the majority of normal human cells and functions to prevent telomere erosion, which causes cellular senescence [49]. Therefore, maintaining telomerase activity in normal cells by modulating hTERT is critical, especially in those who are at risk of premature aging, such as those with obesity. However, in cancer, hTERT might be amplified or mutated, which plays a vital role in the dysregulation of telomerase activity [50]. Due to the limited understanding of the complexity of hTERT regulation by fasting or other diet modulation in aging, obesity, and malignancy, further investigation is required [49,51,52].

An increase in hTERT in this study was expected to correlate with an increase in FOXO3a. These results were expected to be consistent with earlier studies suggesting that FOXO3 can protect telomere shortening in peripheral blood mononuclear cells and is related to higher levels of telomerase activity [43]. However, a significant correlation was only found in the CG. Without intervention, FOXO3a expression correlated with hTERT expression; however, after 10 days of PF treatment, the rise in hTERT in our study might have been driven by other causes that required further exploration. In a study of human fibroblasts, FOXO3a can delay senescence via the SIRT1–FOXO3a–hTERT pathway that activates the transcription of c-MYC [42]. Therefore, this pathway may provide a strong biological theory for understanding the mechanism of FOXO3a in increasing hTERT expression.

Although not as high as in the PFG, hTERT expression in the CG was shown to be elevated. This might be related to increased adiposity, as shown by slight increases in BW, BMI, HC, AC, BF, and VFL in CG participants (Table 1). hTERT mRNA levels were found to be linked with BMI. A study found that leptin levels, an obesogenic factor, were associated with hTERT expression [53]. Leptin promotes hTERT transcription by binding STAT3 and Myc/Max/Mad network proteins to the hTERT promoter [51,53].

This study’s insufficient results, particularly FOXO expression, could be attributed to study limitations. The study sample is relatively small, and the period of fasting, which is only carried out in the minimal metabolic range (12 h), is one factor that requires attention. As a result, further studies must be conducted with more samples, as well as larger and longer studies, to generalize the findings. The metabolic transition occurs typically between 12 and 36 h after food consumption stops, depending on the individual’s liver glycogen concentration at the start of the fast and the amount of energy expenditure/exercise performed throughout the fast, so studies on longer or different fasting durations are required to determine the ideal fasting duration [19].

FOXO3a and hTERT are two genes that play essential roles in aging. Various stimuli regulate these genes and involve multiple pathways [15,52]. Aside from that, obesity and aging are phenomena affected by various causes and complex pathophysiology. As a result, this study has the potential to be expanded by examining both the upstream and downstream pathways of FOXO3a and hTERT, as well as various factors involved in obesity and other aging hallmarks [24]. The lifestyle modification approach for overcoming obesity is not about diet management; further study must be conducted in combination with physical activity and other daily behaviors to improve biomolecular parameters and other physiological parameters such as body composition [54]. However, this study provides new evidence that PF can elevate lifespan-related genes. Therefore, PF as a diet modification can be recommended as an adjuvant strategy to overcome obesity and prevent premature aging safely and cost-effectively, especially in subjects that can adopt another lifestyle approach.

## 5. Conclusions

PF regimens using the 12 h time-restricted eating approach evaluated here could increase the expression of longevity-related genes, including FOXO3a and hTERT in young men with overweight or obesity, as well as vital signs, anthropometry, and body composition. This feature makes PF an intriguing strategy for preventing obesity-induced premature aging, as well as promoting healthy aging.

## Figures and Tables

**Table 1:** Effects of PF on vital signs, and anthropometric and body composition in the CG and PFG.

Parameters	CG (*n* = 19)			PFG (*n* = 17)			*p*-Value of the Difference Parameters between Groups (Δ)
	Pre-Test	Post-Test	*p*-Value	Pre-Test	Post-Test	*p*-Value	
SBP (mmHG)	125.26 (±10.07)	123.05 (±10.37)	^†^ 0.32	121.06 (±9.03)	117.06 (±9.30)	^‡^ 0.02 *	^⸼^ 0.51
DBP (mmHG)	86.32 (±10.65)	84.74 (±8.41)	^‡^ 0.38	85.88 (±7.95)	82.35 (±9.70)	^†^ 0.19	^⸼^ 0.40
HR (x/minute)	83.95 (±12.40)	86.37 (±14.51)	^†^ 0.46	87.59 (±8.59)	87.06 (±13.04)	^†^ 0.88	^⸸^ 0.54
RR (x/minute)	18.84 (±1.54)	18.84 (±1.80)	^‡^ 1.00	18.12 (±2.29)	18.47 (±2.29)	^†^ 0.53	^⸸^ 0.80
BW (kg)	85.42 (±14.82)	85.81 (±14.67)	^†^ 0.18	82.85 (±10.22)	80.44 (±10.77)	^†^ 0.00 *	^⸸^ 0.00 *
BH (m)	170.05 (±6.62)	-	-	169.59 (±6.60)	-	-	-
BMI (kg/m^2^)	29.57 (±5.17)	29.85 (±4.94)	^‡^ 0.17	28.69 (±2.19)	27.88 (±2.42)	^†^ 0.00 *	^⸼^ 0.00 *
HC (cm)	98.37 (±10.10)	98.55 (±10.11)	^‡^ 0.55	99.21 (±10.25)	93.91 (±9.77)	^†^ 0.76	^⸼^ 0.26
AC (cm)	96.87 (±11.36)	96.92 (±11.57)	^‡^ 0.97	94.62 (±7.62)	92.76 (±7.58)	^†^ 0.00 *	^⸼^ 0.02 *
WC (cm)	90.53 (±9.01)	90.45 (±9.52)	^†^ 0.88	88.15 (±6.89)	86.18 (±6.22)	^†^ 0.00 *	^⸸^ 0.02 *
UAC (cm)	33.42 (±3.89)	32.87 (±3.62)	^‡^ 0.13	32.56 (±2.38)	32.56 (±2.37)	^‡^ 8.86	^⸼^ 0.38
BF (%)	25.64 (±5.47)	25.87 (±5.36)	^†^ 0.42	26.57 (±3.34)	26.03 (±3.42)	^‡^ 0.16	^⸼^ 0.09
TBW (%)	49.68 (±4.60)	49.40 (±4.40)	^†^ 0.46	47.80 (±3.52)	47.48 (±3.44)	^†^ 0.43	^⸸^ 0.99
MM (kg)	57.88 (±6.80)	58.54 (±6.02)	^‡^ 0.81	56.57 (±6.58)	55.65 (±6.65)	^†^ 0.00 *	^⸼^ 0.02 *
BM (kg)	3.19 (±0.34)	3.19 (±0.32)	^‡^ 1.00	3.08 (±0.35)	3.05 (±0.34)	^‡^ 0.06	^⸼^ 0.43
VFL (level)	10.91 (±3.13)	10.96 (±3.04)	^‡^ 0.63	10.62 (±1.85)	10.41 (±1.96)	^‡^ 0.02 *	^⸼^ 0.15

Systolic blood pressure (SBP), diastolic blood pressure (DBP), heart rate (HR), respiratory rate (RR), body weight (BW), body height (BH), body mass index (BMI), hip circumference (HC), abdominal circumference (AC), waist circumference (WC), upper arm circumference (UAC), body fat (BF), total body water (TBW), muscle mass (MM), bone mass (BM), visceral fat level (VFL). Data were reported as mean ± standard deviation. The analysis was determined based on the 10-day observation points before (pre-test) and after PF (post-test). The difference between pre-test and post-test data was analyzed using the paired *t*-test (^†^) or Wilcoxon test (^‡^). Delta (Δ) between both groups was determined using the independent *t*-test (^⸸^) or Mann–Whitney U-test (^⸼^). (*) indicates a significant difference.

## Data Availability

The raw data can be obtained via request to the corresponding author.
